# Utility of melatonin in mitigating ionizing radiation-induced testis injury through synergistic interdependence of its biological properties

**DOI:** 10.1186/s40659-022-00401-6

**Published:** 2022-11-04

**Authors:** Maggie E. Amer, Azza I. Othman, Hajer Mohammed Abozaid, Mohamed A. El-Missiry

**Affiliations:** Faculty of Science, Mansoura University, Mansoura, Egypt

**Keywords:** Melatonin, Ionizing radiation, Apoptosis, Inflammation, Sex hormones, Androgen receptors

## Abstract

**Background:**

Ionizing radiations (IR) have widespread useful applications in our daily life; however, they have unfavorable effects on reproductive health. Maintaining testicular health following IR exposure is an important requirement for reproductive potential. The current study explored the role of melatonin (MLT) in mitigating IR-induced injury in young adult rat testis.

**Methods:**

Rats were given daily MLT (25 mg/kg) for 3 and 14 days after receiving 4 Gy γ-radiation.

**Results:**

Serum MLT levels and other antioxidants, including glutathione content, and the activity of glutathione peroxidase and glutathione reductase in the testis of the irradiated rats were remarkably maintained by MLT administration in irradiated rats. Hence, the hydrogen peroxide level declined with remarkably reduced formation of oxidative stress markers, 4-hydroxynonenal, and 8-Hydroxy-2′-deoxyguanosine in the testis of irradiated animals after MLT administration. The redox status improvement caused a remarkable regression of proapoptotic protein (p53, Cyto-c, and caspase-3) in the testis and improved inflammatory cytokines (CRP and IL-6), and anti-inflammatory cytokine (interleukin IL-10) in serum. This is associated with restoration of disturbed sex hormonal balance, androgen receptor upregulation, and testicular cell proliferation activity in irradiated rats, explaining the improvement of sperm parameters (count, motility, viability, and deformation). Consequently, spermatogenic cell depletion and decreased seminiferous tubule diameter and perimeter were attenuated by MLT treatment post irradiation. Moreover, the testis of irradiated-MLT-treated rats showed well-organized histological architecture and normal sperm morphology.

**Conclusions:**

These results show that radiation-induced testicular injury is mitigated following IR exposure through synergistic interdependence between the antioxidant, anti-inflammatory, anti-apoptotic, and anti-DNA damage actions of MLT.

## Introduction

Ionizing radiation (IR) is unavoidable with benefits and risk outcomes. The association between radiation and reproductive health is a prevalent issue without effective and safe treatment. Relevant data concerning radiation-induced testicular injury for overcoming these concerns are limited [1]. The effect of IR on male fertility was studied in terms of spermatogenesis which reported extensive impairment [2]. Thus, finding an efficient treatment agent to preserve fertility is necessary to maximize radiation application to meet global public and professional health needs.

The testis is one of the most radiosensitive organs, and spermatogenic cells are the most sensitive cellular element to IR and non-IR due to high proliferation activity [3]. Recent study report that IR disrupted circadian rhythms of reproductive markers, including decreased sperm motility and disrupted clock gene expression in the testis [4]. Radiation exposure might cause infertility by lowering sperm count and testosterone levels by destroying Leydig and spermatogonial stem cells [5]. A previous study showed that IR disrupts redox balance, induces oxidative DNA injury, activates P53, and stimulates inflammation and apoptosis [6]. Radiation with single doses of > 1 Gy might initiate inflammatory reactions associated with oxidative stress and reduced antioxidant capacity [7]. Inflammatory signaling associated with IR might induce testicular damage [8]. Thus, controlling inflammation and oxidative damage is a primary strategy for ameliorating testicular injury after IR exposure.

Till now, testicular injury after IR exposure has no specific or effective modalities [9]. Studies showed that antioxidants exhibited therapy for radiation damage based on radiation-induced oxidative stress. Melatonin (MLT) is a hormone synthesized and released by the pineal gland and regulated by the dark/light cycle [10]. The advantage of MLT compared with conventional antioxidants is represented by its ability to scavenge several types of free radicals, deactivate pro-oxidant enzymes, stimulate the antioxidant enzyme, such as superoxide dismutase and glutathione peroxidase, and attenuate inflammatory responses at several levels after IR exposure, thereby ameliorating the side effect of radiation [11, 12]. Moreover, MLT’s amphipathic nature enables it to cross biological membranes and blood-organ barriers, thereby protecting macromolecules from oxidative damage [13]. MLT directly acts via activating the G-protein-coupled membrane-bound MLT receptors MT1 and MT2 and indirectly with nuclear orphan receptors from the RORα/RZR family [14, 15].

MLT affects the reproductive function of seasonal breeding animals by regulating the secretion of gonadotropin, luteinizing hormone, and testosterone, which promote testicular maturation [16]. MLT also maintains sperm quality, improves sperm motility, and promotes reproductive performance via several mechanisms [17]. It stimulates testosterone synthesis in Leydig cells via regulating testosterone synthesis-related genes [18], modulating different inflammatory mediator expressions, activating cell signaling pathways responsible for its anti-inflammatory activity [19], and regulating mitochondrial function. Moreover, the beneficial effect of MLT might be due to its ability to inhibit mitochondrial damage in injured cells, reduce senescence, and promote anastasis [20]. Anastasis is survival mechanism enables cell recovery from apoptotic lesions and return to its normal active and functioning state [21]. Previous studies reported the radioprotective role of MLT in several organs, including the liver [22], brain [23], lens [24], and several other organs [25]. Several studies evaluated the protective effect of MLT using wide-range MLT doses (10–300 mg/kg), different administration times (immediately: 24 h), and routs of treatment prior-irradiation [26–30], while pharmacological dose is 10 mg/Kg [29]. However, the post-irradiation treatment with MLT to evaluate its mitigating effect is scanty and thus deserves more clarification. Thus, the current study investigated the impact of MLT on the radiation-induced alteration in testicular structure and function, as well as sperm parameters, in rats to gain clear precise insight into the possible treatment role of MLT to rescue male reproductive health after gamma radiation exposure.

## Results

### MLT improved sperm parameters

**Fig. 1:**
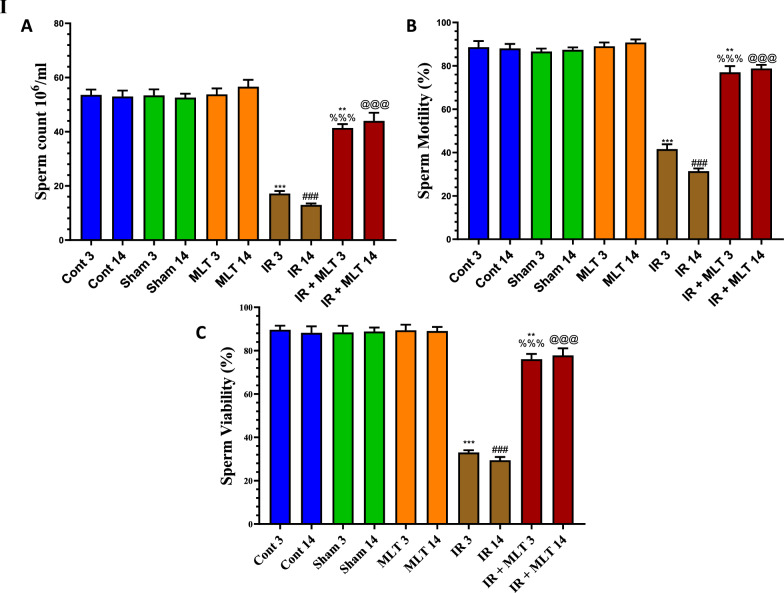

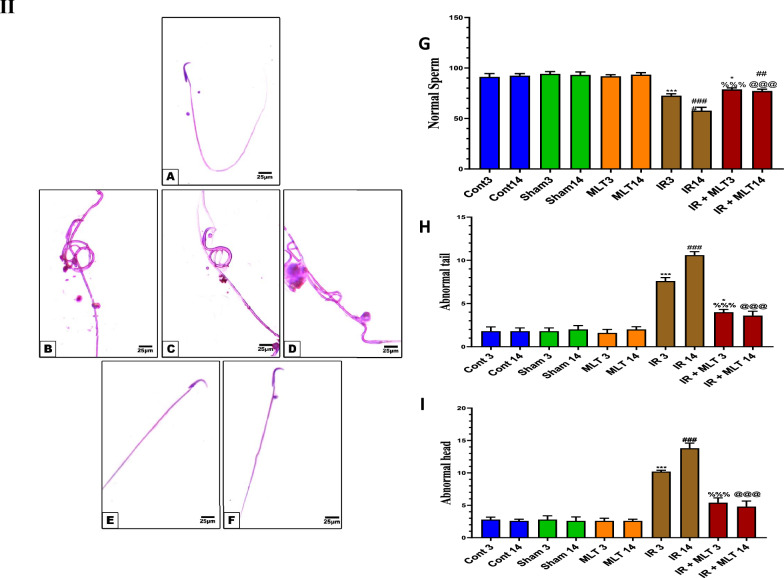
**I** Effect of melatonin (MLT) and irradiation (IR) on the sperm parameters, including **A** sperm count, **B** sperm motility, and **C** sperm viability, in control and different animal groups. Values are expressed as mean ± SEM (n = 5). ** Highly significant at *P* < 0.01*.* ***, ###, %%%, @@@ Very Highly significant at *P* < 0.001*.* **, *** Significant as compared with the control 3 days group. ### Significant as compared with the control 14 days group. %%% Significant as compared with the IR 3 days group. @@@ Significant as compared with the IR 14 days group. Cont: control, MLT: melatonin, IR: irradiated. II Effect of melatonin (MLT) and irradiation (IR) on the morphological classification of rat sperms in the control and different animal groups. **A** Normal control morphology of rat sperm. **B**–**D** Different morphological tail and head abnormalities of rat sperm, respectively, in irradiated rats after 3 and 14 days and **E** and **F** morphology of rat sperm in irradiated rats treated with MLT for 3 and 14 days. (H&E), × 400. **G**–**I** Quantification of normal and abnormal sperm tail and head in the control and different animal groups. Values are expressed as mean ± SEM (n = 5). * Significant at *P* < 0.05*.* ***, ###, %%%, @@@ Very Highly significant at *P* < 0.001. *& *** Significant as compared with the control 3 days group. ### Significant as compared with the control 14 days group. %%% Significant as compared with the IR 3 days group. @@@ Significant as compared with the IR 14 days group. Cont control, MLT melatonin, IR irradiated

Exposure to 4-Gy γ-radiation resulted in morphologically aberrant head and tail after 3 and 14 days of irradiation with a highly significant (*P* < 0.001) drop in sperm count, motility, and viability (Fig. 1) compared to control rats. The daily MLT administration to irradiated rats for 3 and 14 days prevented the disturbed sperm parameters compared to the irradiated animals. The administration of MLT alone did not affect the sperm parameters compared to the control groups.

### MLT ameliorated hormone levels

**Fig. 2:**
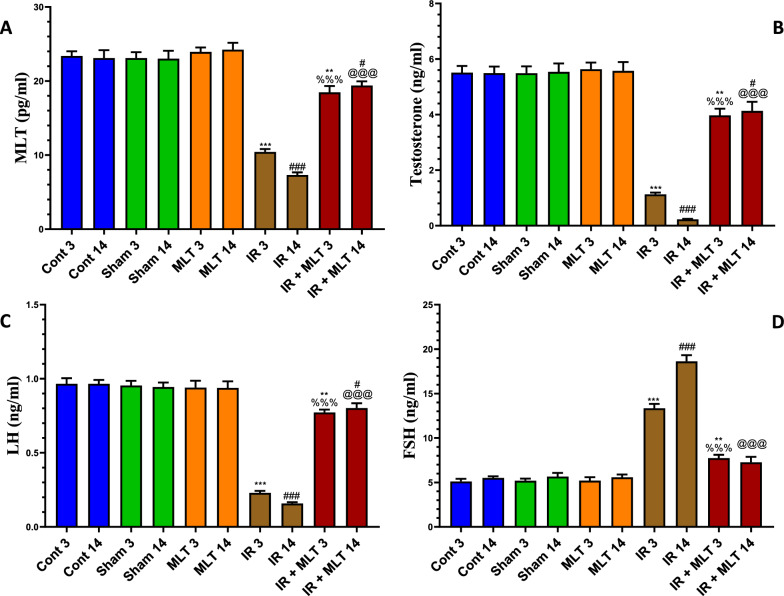
Effect of melatonin (MLT) and irradiation (IR) on serum **A** melatonin (MLT), **B** testosterone, **C** luteinizing hormone (LH), and **D** follicles stimulating hormone (FSH) levels in the control and different animal groups. Values are expressed as mean ± SEM (n = 5). # Significant at *P* < 0.05. ** Highly significant at *P* < 0.01. ***, ###, %%%, @@@ Very Highly significant at *P* < 0.001. **& *** Significant as compared with the control 3 days group. #,### Significant as compared with the control 14 days group. %%% Significant as compared with the IR 3 days group. @@@ Significant as compared with the IR 14 days group. Cont control, MLT melatonin, IR irradiated

Significantly (*P* < *0.001*) decreased MLT, testosterone, and LH were found, while significantly (*P* < *0.001*) increased FSH levels were found in the serum of irradiated rats after 3 and 14 days of irradiation compared with the control rats (Fig. 2). Conversely, oral MLT treatment of irradiated rats for 3 and 14 days significantly normalized the serum hormone levels compared with the irradiated animals to comparable levels of the control groups. The sham and MLT-treated groups showed an insignificant change compared with the control groups (Fig. 2).

### MLT mitigated histopathological and histomorpholoical alterations in the testes of irradiated rats

**Fig. 3:**
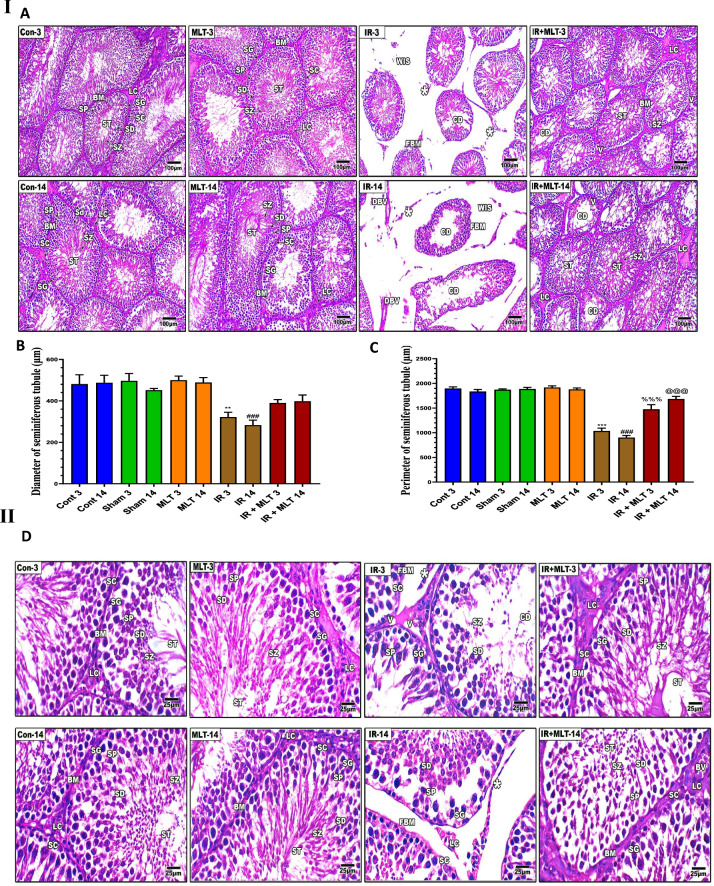
**I and II** Histopathological changes in rat’s testes of the control and different treatment groups after 3 and 14 days of irradiation and melatonin treatment (**A** and **D**). Testicular section of the normal control (**Con-3 and 14**) and MLT-treated rats (**MLT-3 and 14**) showing well-developed seminiferous tubules (ST) enclosed by an intact basement membrane (BM), regular arrangement of germinal epithelium spermatogonia (SG), Spermatocyte (SP), spermatids (SD), spermatozoa (SZ) filling the tubular lumen, Sertoli cell (SC), and prominent interstitial cellularity Leydig cell (LC). Testicular sections of irradiated rats (**IR-3 and 14**) illustrate irregular seminiferous tubule appearance with folded basement membrane (FBM) with detached basement membranes (asterisk), widen interstitial space (WIS), dilation of blood vessels (DBV), degenerated and poorly developed LC, and cell debris in the lumen (CD). Treatment of irradiated rats with MLT (**IR + MLT-3 and 14**) displaying seminiferous tubule and spermatogenesis amelioration in most of the seminiferous tubules with minor cell debris and vacuolation (V). (H&E, ×  = 100 and 400 respectively). Quantification is expressed as the diameter and perimeter of seminiferous tubules (µm) in all studied groups (**B** and **C**, respectively). Each value represents the mean ± SEM of five microscopic fields/tissue samples. ** Highly significant at *P* < 0.01. ***, ###, %%%, @@@ Very Highly significant at *P* < 0.001. **& *** Significant as compared with the control 3 days group. ### Significant as compared with the control 14 days group. %%% Significant as compared with the IR 3 days group. @@@ Significant as compared with the IR 14 days group. Cont control, MLT melatonin, IR irradiated

Testicular sections of the control and MLT-treated rats exhibited normal architecture of seminiferous tubules with typical and active spermatogenic cell layers and spermatozoa. The constant organization and shape of seminiferous tubules were identified by histomorphometrically assessing the diameter and perimeter of seminiferous tubules (Fig. 3).

The irradiated rats displayed mild degenerative changes in the seminiferous tubule with a spermatogenic cell reduction after 3 days post-irradiation**.** Whereas, severe seminiferous tubule necrosis with shrinkage, disorganized, less compact tubule wall, vacuolation of the seminiferous epithelium, absence of spermatogenic cells, and poorly developed Leydig cells were noticed in the testes after 14 days of irradiation**.** Histomorphometrically, the testes of irradiated rats revealed a significant (*p* < *0.001*) reduction in the diameter and perimeter of seminiferous tubules with limited sperms compared with the control groups 3- and 14-days post-irradiation.

The MLT treatment of irradiated rats for 3 days improved the histopathological alteration caused by seminiferous tubule irradiation. However, the MLT treatment of irradiated rats for 14 days markedly ameliorated radiation-induced histopathological effects in which seminiferous tubules showed an increased germinal cell population with active spermatogenesis. These effects were verified by a significant (*p* < *0.001*) amelioration of seminiferous tubule diameter and perimeter compared with the irradiated groups (Fig. 3).

### MLT maintained androgen receptor AR and PCNA expression in the testis

**Fig. 4:**
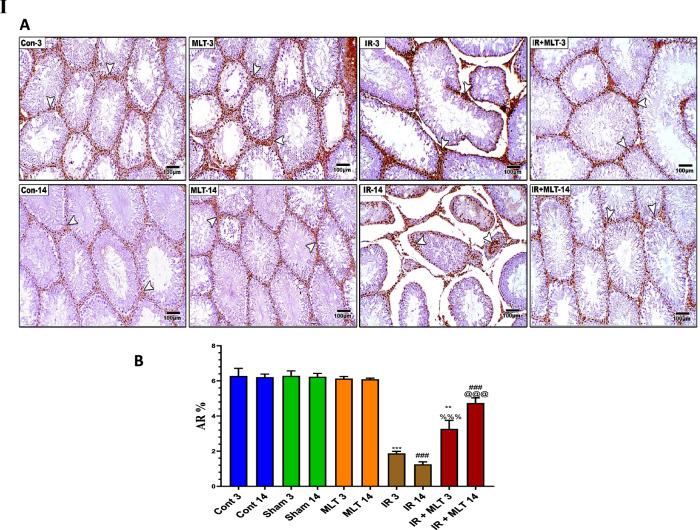

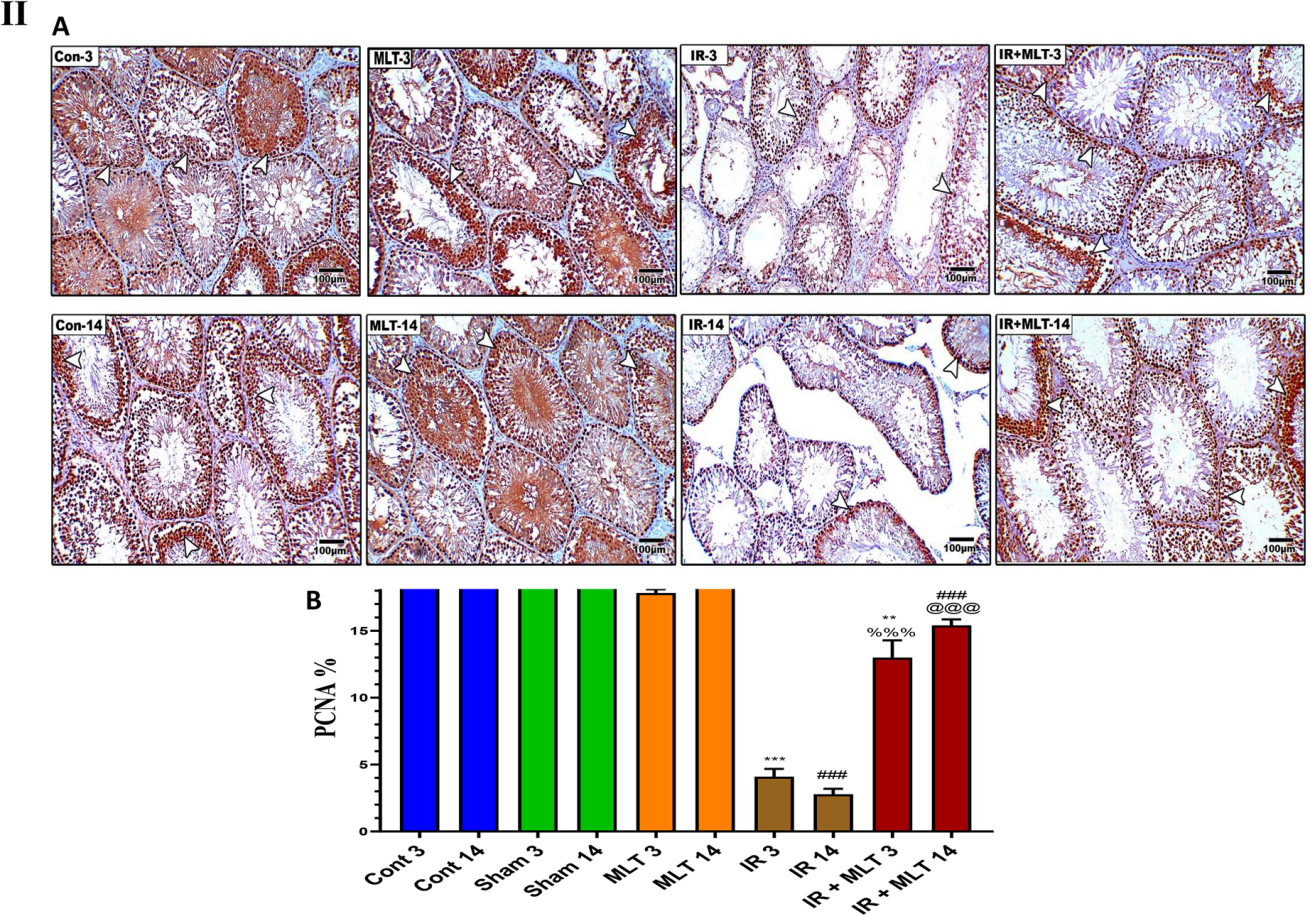
**I A** Immunoassay of androgen receptor (AR) in the testis of the control (**Con-3 and 14**) and MLT-treated rats (**MLT-3 and 14**), revealing strong AR immunostaining within germ cells (arrows). Testicular sections of irradiated rats (**IR-3 and 14**) illustrate mild AR expression in the testis of irradiated rats (arrows) and MLT-treated irradiated rats for 3 and 14 days (**IR + MLT-3 and 14**) show a marked AR immuno-expression amelioration to almost normal (arrows) within germ cells, (IHC × 100). **B** The histogram illustrates the quantification of the expression levels of AR in the various treatment groups. Values are expressed as the means ± SEM of 5 microscopic fields/tissue samples. ** Highly significant at *P* < 0.01. ***, ###, %%%, @@@ Very Highly significant at *P* < 0.001. **& *** Significant as compared with the control 3 days group. ### Significant as compared with the control 14 days group. %%% Significant as compared with the IR 3 days group. @@@ Significant as compared with the IR 14 days group. Cont: control, MLT: melatonin, IR: irradiated. **II** Immunostained testis sections with PCNA of the control (**Con-3 & 14**) and MLT-treated rats (**MLT-3 and 14**), revealing diffused PCNA immuno-expression within seminiferous tubules (arrows). Testicular sections of irradiated rats (**IR-3 and 14**) illustrating slight PCNA expression in the testis of the irradiated rats (arrows) and MLT-treated irradiated rats for 3 and 14 days (**IR + MLT-3 and 14**), showing a marked PCNA immuno-expression amelioration to almost normal (arrows) within the seminiferous tubules, (IHC × 100). **(B)** Histogram illustrating the quantification of the PCNA expression levels in various treatment groups. Values are expressed as the means ± SEM of 5 microscopic fields/tissue samples. ** Highly significant at *P* < 0.01. ***, ###, %%%, @@@ Very Highly significant at *P* < 0.001. **& *** Significant as compared with the control 3 days group. ### Significant as compared with the control 14 days group. %%% Significant as compared with the IR 3 days group. @@@ Significant as compared with the IR 14 days group. Cont control, MLT melatonin, IR irradiated

IHC assessment of the testicular sections from the control and MLT-treated rats exhibited significant (*P* < *0.001*) AR and PCNA immunoexpression within seminiferous tubular cells (Fig. 4). Conversely, the testicular sections of irradiated rats displayed a significant reduction (*P* < *0.001*) in AR and PCNA expression compared with that of the control groups after 3 and 14 days of irradiation. Whereas, the MLT treatment of irradiated rats for 3 and14 days after radiation exposure showed a significant increase (*P* < *0.001*) in AR and PCNA immunoexpression compared to the irradiated groups (Fig. 4). Moreover, the amelioration was greater after MLT treatment for 14 days than 3 days.

### MLT normalized apoptosis-regulating proteins

**Fig. 5:**
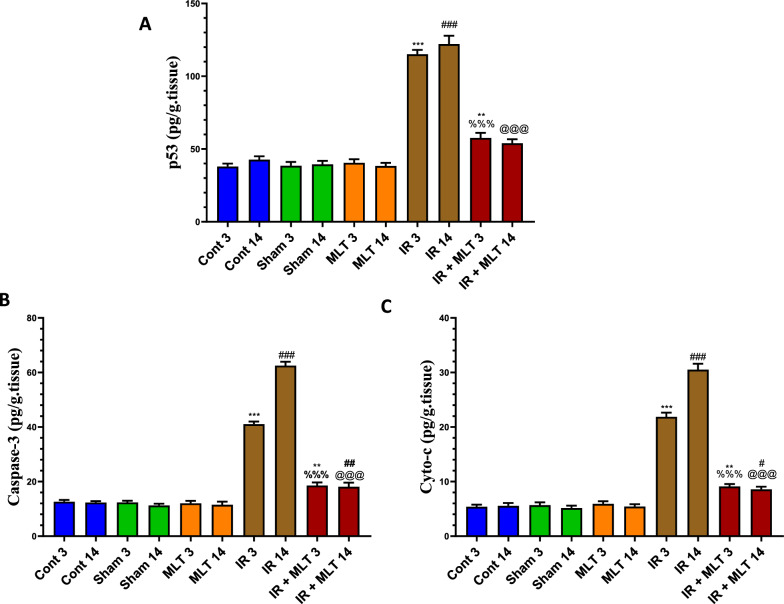
Effect of melatonin (MLT) and irradiation (IR) on the levels of apoptotic proteins, **A** p53, **B** caspase-3, and **C** cytochrome-c (Cyto-c) in the testes in the control and different animal groups. Values are expressed as mean ± SEM (n = 5). # Significant at *P* < 0.05. ** Highly significant at *P* < 0.01. ***, ###, %%%, @@@ Very Highly significant at *P* < 0.001. **& *** Significant as compared with the control 3 days group. #, ### Significant as compared with the control 14 days group. %%% Significant as compared with the IR 3 days group. @@@ Significant as compared with the IR 14 days group. Cont control, MLT melatonin, IR irradiated

The levels of apoptotic proteins, including p53, cytochrome-c (Cyto-c), and caspase-3 were significantly (*P* < *0.001*) upregulated in the testes of irradiated rats after 3 and 14 days of irradiation compared with the control animals. Conversely, the MLT treatment of irradiated rats for 3 and14 days significantly (*P* < *0.001*) decreased the levels of p53, Cyto-c, and caspase-3 compared with the irradiated groups (Fig. 5). The sham and MLT-treated groups showed an insignificant change in these proteins compared with the control groups.

### MLT prevented DNA injury in the testes of irradiated rats

**Fig. 6:**
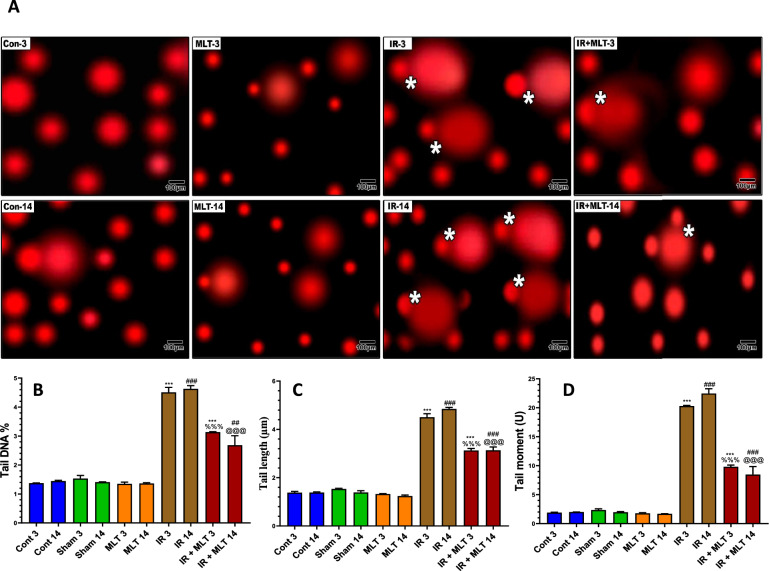
Effect of melatonin (MLT) and irradiation (IR) on the percentage of DNA damage in the testes of rats in different groups using the comet assay technique. **A** The appearance of the microscopic images of representative comets for the different groups is shown. **B** Percentage of tail DNA, **C** tail length, and **D** tail moment. The values are expressed as the means ± SEM (n = 5). ** Highly significant at *P* < 0.01. ***, ###, %%%, @@@ Very Highly significant at *P* < 0.001. **& *** Significant as compared with the control 3 days group. ### Significant as compared with the control 14 days group. %%% Significant as compared with the IR 3 days group. @@@ Significant as compared with the IR 14 days group. Cont control, MLT melatonin, IR irradiated

The levels of DNA comet parameters in the testes of the experimental groups are displayed in Fig. 6. Irradiated rats displayed significantly (*P* < *0.001*) increased levels of all comet attributes, including tail DNA%, tail length, and tail moment, compared with control animals. Meanwhile, the MLT treatment of irradiated rats for 3- and 14-days post-radiation exposure significantly (*P* < *0.001*) suppressed the increased comet parameters compared with the irradiated groups. An insignificant change was observed in the sham and MLT-treated groups compared with the control groups.

### MLT improved pro-inflammatory and anti-inflammatory cytokines

**Fig. 7:**
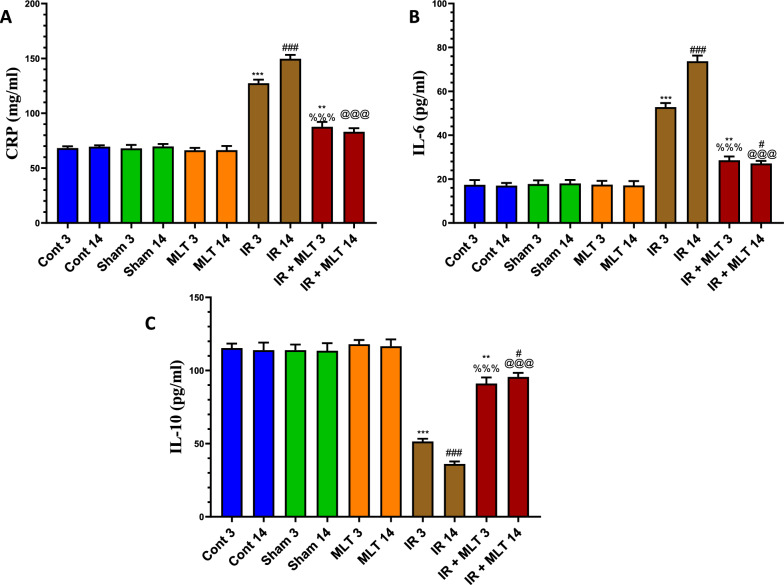
Effect of melatonin (MLT) and irradiation (IR) on serum levels of the inflammatory cytokines, **A** C-reactive protein (CRP) and **B** IL-6, as well as the anti-inflammatory cytokine **C** IL-10, in various treatment groups. Values are expressed as mean ± SEM (n = 5). ** Highly significant at *P* < 0.01. ***, ###, %%%, @@@ Very Highly significant at *P* < 0.001. **& *** Significant as compared with the control 3 days group. #, ### Significant as compared with the control 14 days group. %%% Significant as compared with the IR 3 days group. @@@ Significant as compared with the IR 14 days group. Cont control, MLT melatonin, IR irradiated

Exposure to γ-radiation resulted in a significant (*P* < *0.001*) surge of inflammatory cytokines, including CRP and IL-6, with a significant decline in the anti-inflammatory cytokine, IL-10, levels in the serum of the irradiated rats after 3- and 14-days post-irradiation compared with that of the control animals. However, the oral MLT treatment of irradiated rats for 3 and 14 days after radiation exposure caused significantly (*P* < *0.001*) normal inflammatory and anti-inflammatory cytokine concentrations compared with the irradiated animals, with comparable levels with the control groups (Fig. 7). An insignificant change in these cytokines was observed in the sham and MLT-treated animals compared with the control groups.

### MLT prevented oxidative stress and improved the antioxidants levels in the testes

**Fig. 8:**
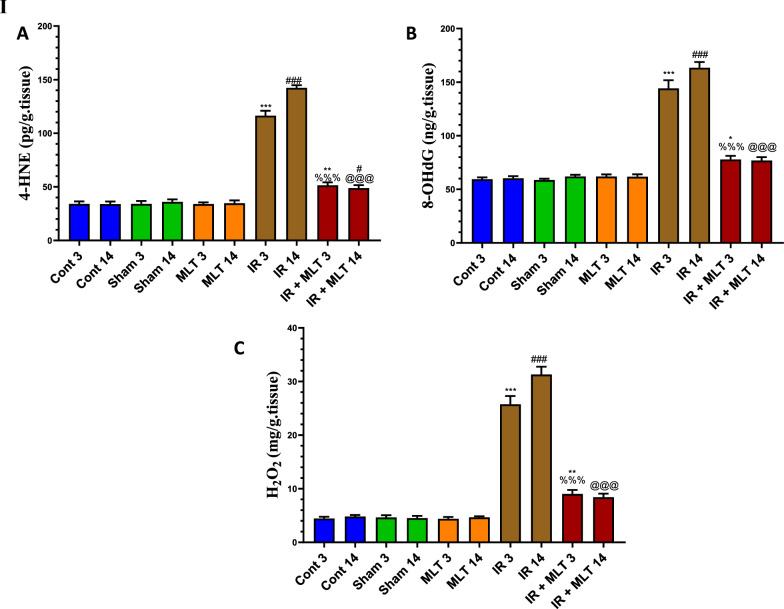

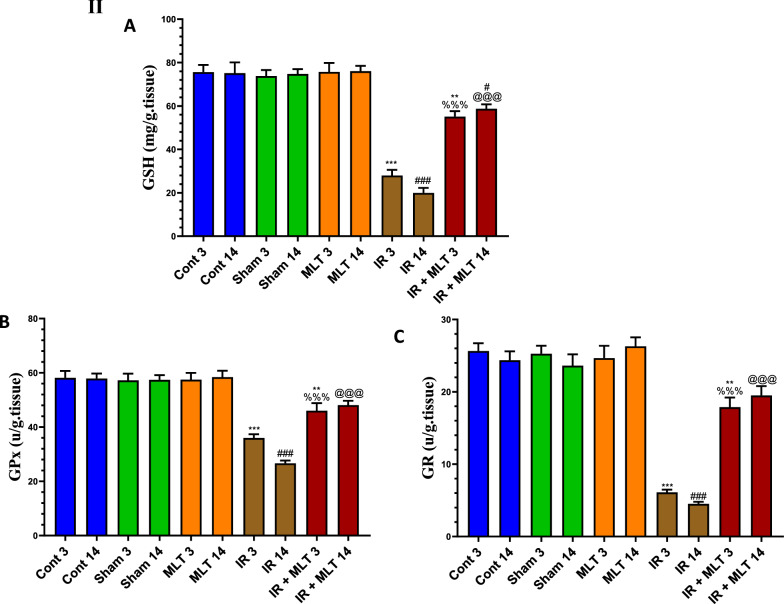
**I** Effect of melatonin (MLT) and irradiation (IR) on the oxidative stress markers, including **A** 4-hydroxynonenal (4-HNE), **B** 8-Hydroxyguanosine (8-OHdG), and **C** hydrogen peroxide (H_2_O_2_) concentrations, in the testicular tissues of rats in the control and different treatment groups. Values are expressed as mean ± SEM (n = 5). ** Highly significant at *P* < 0.01. ***, ###, %%%, @@@ Very Highly significant at *P* < 0.001. **& *** Significant as compared with the control 3 days group. #, ### Significant as compared with the control 14 days group. %%% Significant as compared with the IR 3 days group. @@@ Significant as compared with the IR 14 days group. *Cont* control, *MLT* melatonin, *IR* irradiated. II Effect of melatonin (MLT) and irradiation (IR) on the antioxidants in the testicular tissues of rats in the control and different treatment groups. **A** glutathione (GSH) content, **B** glutathione peroxidase (GPx), and **C** glutathione reductase (GR) activities. Values are expressed as mean ± SEM (n = 5). ** Highly significant at *P* < 0.01. ***, ###, %%%, @@@ Very Highly significant at *P* < 0.001). **& *** Significant as compared with the control 3 days group. #, ### Significant as compared with the control 14 days group. %%% Significant as compared with the IR 3 days group. @@@ Significant as compared with the IR 14 days group. Cont control, MLT melatonin, IR irradiated

Exposure to 4-Gy γ-radiation induced a significantly (*P* < *0.001*) increased oxidative stress represented by elevation of the H_2_O_2_, 4-HNE, and 8-OHdG levels in testes after 3- and 14-days post-exposure compared with the control animals. This effect was accompanied by a highly significantly (*P* < *0.001*) decreased antioxidants, including GSH content and GPx and GR activities in the testes of the irradiated rats. However, MLT therapy for 3 and 14 days considerably (*P* < *0.001*) prevented the rise in 4-HNE, 8-OHdG, and H_2_O_2_ and significantly (*P* < *0.001*) maintained the higher antioxidant levels than those seen in the irradiated rats (Fig. 8I, II). The sham and MLT-treated groups displayed no changes in antioxidant and oxidative stress levels, in contrast to the control groups.

## Discussion

The emerging problem of testicular dysfunction becomes a major health challenge worldwide, considering infertility associated with radiation exposure during radiotherapy, radiation technology application, or accidental radiation overexposure. MLT has been implicated in numerous studies as a radio-protector [23, 29], but its ameliorating impact on testicular dysfunction and structural changes following radiation exposure are rare and imprecise. Thus, the effect of MLT treatment on IR-induced testicular damage and sperm defect was investigated in young adult rats after 3- and 14-days post-irradiation. The current study revealed that MLT treatment mitigated testicular structure and function, as well as improved sperm parameters, through interconnecting biological events, including antioxidant, anti-inflammatory, and anti-apoptotic mechanisms.

The present study revealed that endogenous MLT levels significantly decreased in irradiated rats, indicating pineal gland function and MLT synthesis disruptions. Daily treatment with 25 mg/kg of MLT post-irradiation produced remarkable serum MLT level elevations in the irradiated rats to comparable control levels, signifying pineal gland function improvement in terms of MLT synthesis and release. This agrees with the previous report that daily MLT supplementation improved endogenous MLT levels and prevents oxidative damage in human sperm [31].

The testis is one of the most radio-sensitive organs because of its high proliferative activity [5] which makes it vulnerable to free radical attack and the development of indirect damages. This study revealed that a single whole-body exposure to 4 Gy γ-rays caused remarkable testicular degeneration and dysfunction. These findings are in agreement with a recent study that reported a significant alteration in the histological parameters of seminiferous tubules, such as the numbers of spermatogonia and mature sperms with Leydig cells damage after X- and γ-ray exposures [32, 33]. These changes might be attributed to increased apoptosis represented by proapoptotic protein upregulation due to decreased endogenous antioxidants, including MLT, and uprising oxidative injury, as well as prominent inflammation signified by a surge of inflammatory cytokines in the irradiated rats.

The present results revealed pronounced activation of the intrinsic mitochondrial pathway of apoptosis in irradiated rat testis. Radiation-induced reactive oxygen species (ROS) targeted mitochondria and their electron transport system, resulting in apoptosis via the Cyto-c/caspase-3 pathway [34] and causing pyknosis and karyolysis of germ cells [35]. The current study revealed increased levels of H_2_O_2_, Cyto-c, and caspase-3, with histological alteration development in the testis of irradiated rats. The release of Cyto-c to the cytosol is attributed to dysfunction of mitochondrial membrane induced by radiation-induced Bax upregulation [36, 37]. The current MLT treatment regimen after IR exposure resulted in a remarkable proapoptotic protein regression and consequently, apoptosis. This is designated by a decreased p53, Cyto-c, and caspase-3 with remarkable histological testicular structure improvement, indicating apoptotic inhibition. The effect of MLT on these pro-apoptotic mediators was suggested to play a key role in improving the germinal cell epithelium thickness and the testicular tissue atrophy. The antiapoptotic effect of MLT might be due to its preservation of mitochondrial electron transport chain by multiple ways. This includes its ability to downregulate inducible nitric oxide synthase to reduce the release of nitric oxide within mitochondria and its capacity to improve the intramitochondrial antioxidant defense by enhancing GSH levels and up-regulating the GPx and superoxide dismutase activities in the intermembrane space [35] by a mechanism involving a mitochondrial cytochrome P450 subform [38]. These reports are supported by the present finding that displayed testicular antioxidant improvements, including MLT, and decreased oxidative stress with MLT treatment after irradiation. These findings are congruent with previous reports after IR and heat stressed testis [39, 40], which might clarify the MLT’s ability to preserve the Sertoli cell tight-junction [41, 42], of which the destruction was suggested to cause spermatogenic disruption and thus, male infertility [43].

MLT treatment after irradiation resulted in a significantly decreased H_2_O_2_ in the testis, which agrees with a previous study that showed the ability of MLT to directly eliminate H_2_O_2_ and form an intermediate compound that can be eliminated by CAT [44]. Therefore, we observed a marked decrease in the formation of 4-HNE and 8-OHdG levels in the testis of MLT-treated rats after irradiation, signifying decreased oxidative destruction of testicular cells. MLT replenished the GSH content and GPx and GR activities in the testis, which are important for eliminating free radicals and subsequent inhibition of lipid peroxidation and DNA oxidation in the testis of irradiated rats, in addition to its free radical scavenging ability. GSH is an antioxidant in cytoplasm and mitochondria that scavenges free radicals and regulates cellular redox homeostasis [45]. GSH is essential because Sertoli cells convert GSH to amino acids that are required in spermatogenesis [24]. Similarly, GPx, which catalyzes lipid hydroperoxide elimination, expresses the pivotal link between selenium, sperm quality, and fertility [45]. Moreover, MLT improves neutrophil activity and prevents apoptosis due to the dysfunctional GSH redox system in the testis [46]. Thus, MLT mitigates IR-induced testicular oxidative stress via rejuvenating the GSH redox system by acting as a parallel redox and defense machinery. Moreover, MLT is a potent endogenous antioxidant, strong free radical scavenger, and is involved in the homeostasis of multiple biological functions [47]; thus, MLT level restoration in irradiated rats promoted the antioxidant capacity of the testis and the sperm.

Oxidative DNA injury plays an important role in the development of radiation-induced deleterious effects on testicular cells. This may result in chromosomal aberrations and apoptosis that lead to testicular atrophy, sperm deformation, and infertility [39]. Our results revealed that MLT alleviated the DNA strand breakage, which might be linked to the antiapoptotic effect on testicular cells. This is confirmed by p53 level downregulation after MLT treatment in the irradiated rats. This effect might be related to MLT’s radical scavenging activity and DNA repair mechanisms via activating DNA repair enzymes and de novo protein synthesis associated with DNA repair [48]. The improvement of all comet parameters in MLT-irradiated rats confirms its mitigating effect on DNA and genetic materials following irradiation. Thus, the ability of MLT to maintain DNA integrity might contribute to the improvement of testicular function, structure, and improved sperm parameters.

Moreover, radiation-induced DNA injury induces cell cycle arrest [49] and apoptosis in germ cells via p53 activation that stimulates apoptotic mediators, thereby explaining the increased spermatogonia apoptosis in the previous study [50]. This might elucidate the altered sperm parameters and their absence from seminiferous tubules of irradiated rats in the current study. This is congruent with the study that showed IR increased p53 expression in spermatogonia and spermatocytes in mouse testis [51]. The MLT treatment regulated the p53 activation and revealed lower expression compared with the irradiated rats denoting the favorable effect of MLT on testicular tissues. This effect is attributed to the increased antioxidant status and several pro-apoptotic signaling pathway deactivations, including p21, Bax, Cyto-C, and active caspases-3 [52], and ultimately, regular spermatogenesis [49].

Spermatogonia are characterized by high proliferative activity [53]. The present study revealed a significantly decreased PCNA expression in spermatogonia and primary spermatocytes in irradiated rats, signifying reduced proliferation activity and spermatogenesis. These findings might explain the absence of sperms in seminiferous tubules of the testis of irradiated rats which might be due to incomplete secondary spermatocyte transition to sperm. Conversely, PCNA-positive cells were strongly detected in the testis in the control and IR-MLT-treated rats, indicating that MLT improved the PCNA reactivity and restored proliferation activity post-irradiation. This is supported by the appearance of all lineages of spermatogonial cells and regular sperm parameters in the current study and the amelioration of damage to Sertoli cells after the exposure to X- and γ-rays [28, 32].

Furthermore, the current results revealed significantly decreased testosterone and LH levels with an increased FSH level. The disruption of sex hormone balance explains the decreased sperm count in the irradiated rats, which may correlate with Sertoli cell dysfunction and spermatogenic cell reduction in the testis of the irradiated rats [54]. The low testosterone and LH levels in irradiated rats indicate Leydig cell steroidogenesis dysfunction via LH signal transduction impairment due to the decreased testicular LH receptor number following radiation exposure [55]. The present results revealed a remarkable serum sex hormone levels improvement in MLT-treated irradiated rats, thereby restoring germ-cell and somatic cell population in the testis. These findings are compatible with a recent report that MLT directly affects the testis and testosterone synthesis from Leydig cells in animals [56]. Thus, MLT may promote male reproductive performance and increase testosterone synthesis in mammalian Leydig cells. This action was primarily mediated by the MLT nuclear receptor RAR-related orphan receptor alpha (RORα) because the blockade of this receptor suppressed the effect of MLT on testosterone synthesis [18].

Present results revealed a significant AR expression down-regulation in the testis of irradiated rats. Spermatogenesis and male fertility are significantly influenced by androgens and AR. Testosterone acts through AR, and its signaling in the testis is essential for spermiation [57]. ARs are essential for Leydig and Sertoli cell activities for meiosis completion, thereby accomplishing spermatogenesis [58]. Present results revealed that MLT administration maintained the AR expression within control levels. MLT is the major physiological regulator of seasonal reproduction in middle age and adult mammals; thus, the pineal gland and the testis might be functionally linked [59], suggesting that the relationship between MLT and AR is important for testis physiology. The presence of MLT receptors in all testicular cell types [60] has led us to suggest that MLT maintains healthy testicular cells by protecting AR expression, probably via its antioxidant and scavenging ROS activity.

The testis is an immune privilege organ because of the prominent presence of macrophages within the interstitial compartment of the testis [61]. MLT penetrates the blood-testis barrier and influences the immune system in the testis [62]. IR exposure resulted in severe inflammation evidenced by significantly increased serum IL-6 and CRP levels and decreased IL-10 levels. Chemical agents and free radicals secreted by testicular macrophages during inflammation inhibit the expression of steroid synthesis-related proteins and genes in Leydig cells, which is associated with the down-regulation of testosterone synthesis [12]. The present investigation revealed that MLT prevented the increased pro-inflammatory cytokines and improved the anti-inflammatory cytokine in the serum of the irradiated rats, indicating inflammatory response and tissue damage amelioration. MLT reduces tissue damage during inflammatory reactions through a variety of mechanisms, including firstly, direct scavenging of various ROS and RNS contribute to inflammatory response suppression and associated tissue destruction via cell macromolecular oxidative damage reduction. Moreover, MLT exerted its anti-inflammatory effect by inhabiting the inducible nitric oxide synthase expression and production [63]. Secondly, MLT can modulate the transcriptional activity of nuclear factor kappa B and mitogen-activated protein kinases [19]. These factors translocate to the nucleus and bind to DNA, thereby lowering the activation of a range of proinflammatory cytokines [63]. Thirdly, MLT reduces the generation of adhesion molecules that enhance leukocyte adherence to endothelial cells [64]. Thus, the anti-inflammatory and antioxidant effects of MLT are intertwined [47].

## Conclusions

In conclusion, inflammation amelioration, proapoptotic protein control, and redox balance modification are the main interconnected mechanisms that may contribute to MLT’s positive effects after radiation exposure. The improvement in histological and physiological abnormalities in the testis, as well as the sperm parameters of mice exposed to IR, is reflected and explained by these effects. Therefore, MLT helps preserve male fertility in people subjected to radiotherapy, radiation-based application, or accidental IR overexposures.

## Materials and methods

### Irradiation

Rats were exposed to whole-body γ-radiation at a single dose of 4 Gy delivered at a rate of 695 mGy for 9 min and 23 s. The radiation source was Cs137 (GC-40, Nordion, Canada), at the National Center for Radiation Research and Technology, Egyptian Atomic Energy Authority, Nasr City, Cairo, Egypt.

### Animals

Fifty adults male Wistar rats (8 weeks old) with body weight (BW) of 90–100 g were obtained from the animal house of VACSERA, Cairo, Egypt. Rats were housed in plastic cages with wood-ship bedding renewed daily. All rats were maintained under controlled humidity, temperature (25 ℃), and photoperiod (12-h light/12-h dark). Animals were fed a commercial rodent pellet diet and water ad libitum. The study protocol was approved by the animal ethics committee of the Faculty of Science, Mansoura University, Egypt (approval number, Sci-Z-M-2021–71).

### Experimental design

After a week of acclimatization, the rats were randomly divided into 10 groups, with 5 animals each: 2 **control groups (Cont 3 and 14)** of normal rats without treatment, 2 **sham groups (Sham 3 and 14)** orally administrated saline with gastric tube only for 3 or 14 days, 2 **MLT-treated groups (MLT 3 and 14)** orally administrated MLT (Sigma Co., St. Louis, MO, USA) at a dose of 25 mg/kg BW [65] for 3 or 14 days [29], 2 **irradiated groups (IR 3 and 14)** were exposed to a single dose of gamma radiation (4 Gy) and were sacrificed after 3 and 14 days post-irradiation, and 2 **irradiated + MLT-treated groups (IR + MLT 3 and 14) who were** irradiated and administrated MLT (25 mg/kg BW) with a gastric tube for 3 or 14 days and were sacrificed after 3 and 14 days post-irradiation.

### Sample collection

After 3- and 14-days post-irradiation, overnight fasted rats were anesthetized with ketamine/xylazine (0.1 ml/100 g BW intraperitoneally) [66]. Blood samples were withdrawn by cardiac puncture and placed into non-heparinized tubes to separate serum by centrifugation at 3000 rpm for 15 min and stored at − 20 ℃ until biochemical analysis. Animals were dissected to obtain the testes and cauda epididymis of each rat, and then cleaned and washed with normal physiological saline. Small deep cuts were made along both proximal and distal cauda of each epididymis and then incubated for 5 min at 37 ℃ to release sperm. The sperm suspensions were gently mixed and used immediately for sperm analysis. Left testes were homogenized in chilled Tris–HCl buffer (0.1 M, pH 7.4) using a tissue homogenizer. The supernatant obtained by centrifugation was kept at − 20 ℃ until biochemical analysis. The right testes were fixed in 10% neutral formalin until further processing for histological and immunohistochemical (IHC) studies.

### Sperm analysis

Sperm count was performed using a hemocytometer following the previously-mentioned method [67] after dilution 100 times with fresh medium. Sperm viability was determined using eosin-nigrosin stains (one part of 5% bluish eosin solution, Carl Roth Gmbh + Co. KG, Germany) to four parts of 10% nigrosin aqueous solution (Sigma-Aldrich, USA))[68]. A sperm smear was prepared on a glass slide and stained with hematoxylin and eosin to examine sperm abnormalities [69]. All slides were observed under a light microscope at 400 × magnification and photographed using Olympus light microscope with a camera (Amscope MU1000).

### Biochemical analysis

Serum MLT levels were determined using the enzyme-linked immunosorbent assay (ELISA) kit (Fine Test, Wuhan Biotech, China, (**Catalog # ER1169**) following the manufacturer’s instructions. The testosterone was estimated in serum by ELISA techniques following the manufacturer’s instructions using kits obtained from Crystal Chem Inc., USA (**Catalog # 80,550**). Furthermore, serum follicle-stimulating hormone (**Catalog #E-EL-R0391**) and Luteinizing hormone (LH) (**Catalog #E-EL-R0026**) concentrations were assayed using ELISA kits (Elabscience Biotechnology Company, Beijing, China) following the manufacturer’s instructions.

Cytokines levels were estimated by ELISA kits, provided by My Biosource (San Diego, USA), according to the instruction manual for interleukin-6 (IL-6) (**Catalog # MBS269892**), IL-10 (**Catalog# MBS034393**), and C-reactive protein (CRP) (**Catalog# MBS453159**).

In testes homogenate, 4-hydroxynonenal (4-HNE) (**Catalog#** ER1587) and 8-hydroxy-2' -deoxyguanosine (8-OHdG) (**Catalog#** EU2548) were estimated using Fine Test, (Wuhan Biotech, China), while hydrogen peroxide (H_2_O_2_) (**Catalog#** MBS3808898) purchased from My Biosource (San Diego, USA) was estimated following the manufacturer’s instructions. The glutathione (GSH) (**Catalog#** MBS1600118) levels and glutathione peroxidase (GPx) (**Catalog#** MBS744364) and glutathione reductase (GR) (**Catalog#** MBS9308239) activities in the testis homogenates were estimated using My Biosource (San Diego, USA) ELISA kits following the manufacturer’s instructions.

The quantitative determination of p53 (**Catalog# MBS723886**), cytochrome-c (**Catalog#** MBS727663), and caspase-3 (**Catalog#** MBS743552) concentration in the testis homogenates were determined by ELISA technique following the instruction of the kits obtained from My Biosource (San Diego, USA).

### Comet assay

DNA damage in the testes was assessed using the single-cell gel electrophoresis (comet assay) method [70]. The DNA strand breaks quantification in the obtained images was performed with CASP software to directly obtain the DNA percentage in the tail, as well as the tail length and moment.

### Histological examination of liver sections

Fixed testes were dehydrated and cleared, and then embedded in paraffin wax. Sections (5-μm) were prepared and stained with hematoxylin and eosin (H&E) following routine protocol and were examined using a light microscope and photographed using Olympus light microscope with a camera (Amscope MU1000). Seminiferous tubule perimeter and diameter were digital histomorphometrically analyzed using computer-assisted digital image J software.

### IHC analysis of androgen receptor (AR) and Proliferating cell nuclear antigen (PCNA)

Paraffin-embedded testicular sections were deparaffinized in xylene and then processed for IHC staining using the labeled streptavidin–biotin immunoperoxidase technique [71]. Briefly, sections were incubated overnight at 4 °C with primary antibodies, including rabbit polyclonal anti-AR antibody (**Cat #** PA1-110) and mouse monoclonal anti-PCNA antibody (Cat # 13–3900) (ThermoFisher Scientific, USA). The dilution was 1:20 for AR and 1:100 for PCNA following the manufacturer’s instructions. The labeling index was assessed as previously described [72] using Image J software.

### Statistical analysis

GraphPad Prism 7.02 software was used for statistical analysis after checking the normality distribution for each group. Results were expressed as mean ± standard error of the mean (SEM) (n = 5). Statistical comparisons were evaluated by a one-way analysis of variance followed by Duncan’s multiple range tests.

## Data Availability

All data generated or analyzed during this study are included in this published article.
