# Correction: Utility of melatonin in mitigating ionizing radiation‑induced testis injury through synergistic interdependence of its biological properties

**DOI:** 10.1186/s40659-023-00447-0

**Published:** 2023-07-12

**Authors:** Maggie E. Amer, Azza I. Othman, Hajer Mohammed Abozaid, Mohamed A. El‑Missiry

**Affiliations:** Faculty of Science, Mansoura University, Mansoura, Egypt

**Correction: Biological Research (2022) 55:33** 10.1186/s40659-022-00401-6

Following publication of the original article [1], the authors identified an error in Figs. 3D and 4. The correct version of Figs. 3D and 4 are provided in this correction.

**Fig. 3:**
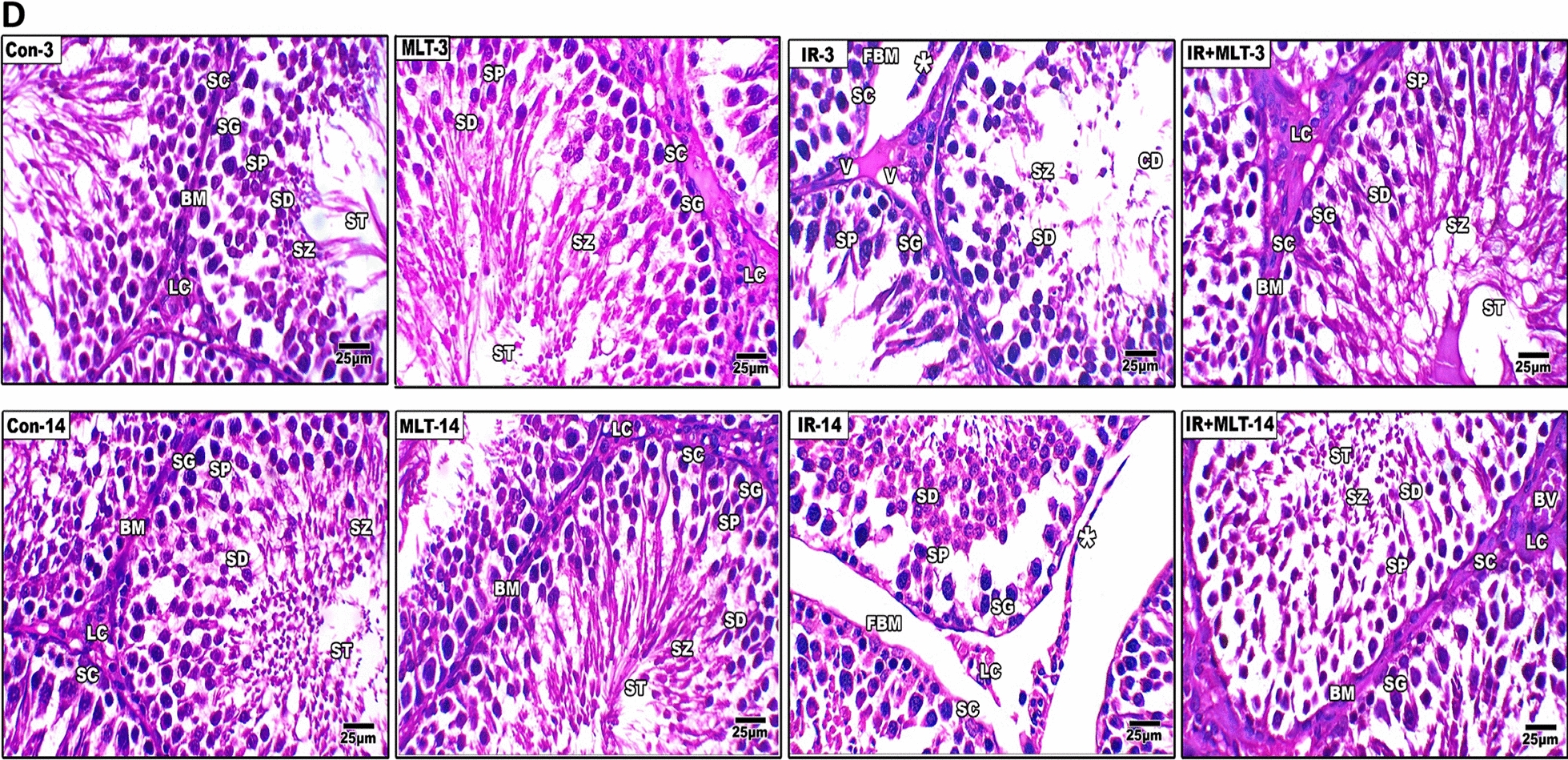
**I** and **II** Histopathological changes in rat’s testes of the control and different treatment groups after 3 and 14 days of irradiation and melatonin treatment (**A** and **D**). Testicular section of the normal control (Con-3 and 14) and MLT-treated rats (MLT-3 and 14) showing well-developed seminiferous tubules (ST) enclosed by an intact basement membrane (BM), regular arrangement of germinal epithelium spermatogonia (SG), Spermatocyte (SP), spermatids (SD), spermatozoa (SZ) filling the tubular lumen, Sertoli cell (SC), and prominent interstitial cellularity Leydig cell (LC). Testicular sections of irradiated rats (IR-3 and 14) illustrate irregular seminiferous tubule appearance with folded basement membrane (FBM) with detached basement membranes (asterisk), widen interstitial space (WIS), dilation of blood vessels (DBV), degenerated and poorly developed LC, and cell debris in the lumen (CD). Treatment of irradiated rats with MLT (IR + MLT-3 and 14) displaying seminiferous tubule and spermatogenesis amelioration in most of the seminiferous tubules with minor cell debris and vacuolation (V). (H&E, ×  = 100 and 400 respectively). Quantification is expressed as the diameter and perimeter of seminiferous tubules (μm) in all studied groups (**B** and **C**, respectively). Each value represents the mean ± SEM of five microscopic fields/tissue samples. ** Highly significant at *P* < 0.01. ***, ###, %%%, @@@ Very Highly significant at *P* < 0.001. ** & *** Significant as compared with the control 3 days group. ### Significant as compared with the control 14 days group. %%% Significant as compared with the IR 3 days group. @@@Significant as compared with the IR 14 days group. *Cont* control, *MLT* melatonin, *IR* irradiated

**Fig. 4:**
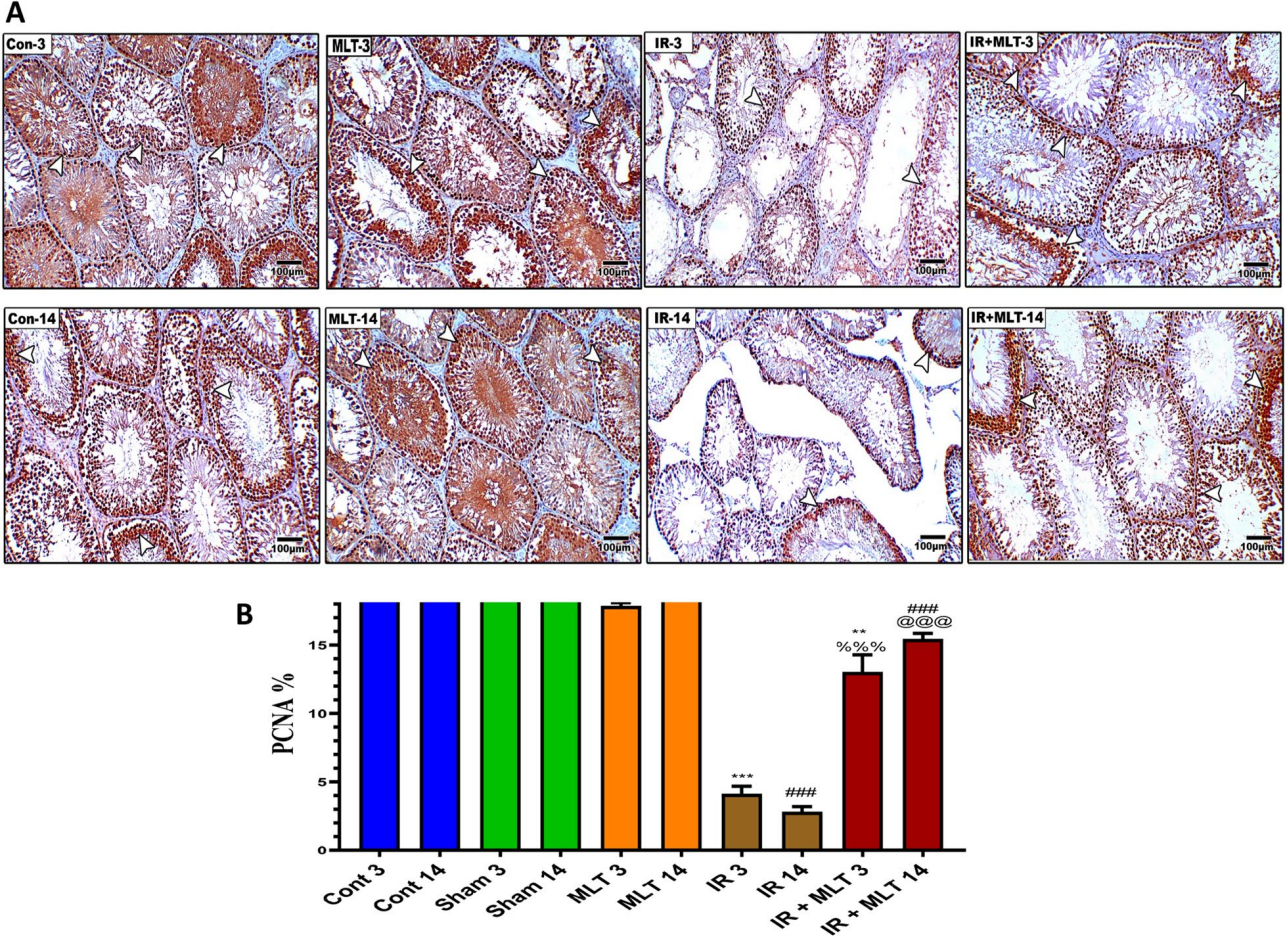
**I A** Immunoassay of androgen receptor (AR) in the testis of the control (Con-3 and 14) and MLT-treated rats (MLT-3 and 14), revealing strong AR immunostaining within germ cells (arrows). Testicular sections of irradiated rats (IR-3 and 14) illustrate mild AR expression in the testis of irradiated rats (arrows) and MLT-treated irradiated rats for 3 and 14 days (IR + MLT-3 and 14) show a marked AR immuno-expression amelioration to almost normal (arrows) within germ cells, (IHC × 100). **B** The histogram illustrates the quantification of the expression levels of AR in the various treatment groups. Values are expressed as the means ± SEM of 5 microscopic fields/tissue samples. ** Highly significant at *P* < 0.01. ***, ###, %%%, @@@ Very Highly significant at *P* < 0.001. **& *** Significant as compared with the control 3 days group. ### Significant as compared with the control 14 days group. %%% Significant as compared with the IR 3 days group. @@@ Significant as compared with the IR 14 days group. Cont: control, MLT: melatonin, IR: irradiated. **II** Immunostained testis sections with PCNA of the control (Con-3 & 14) and MLT-treated rats (MLT-3 and 14), revealing diffused PCNA immuno-expression within seminiferous tubules (arrows). Testicular sections of irradiated rats (IR-3 and 14) illustrating slight PCNA expression in the testis of the irradiated rats (arrows) and MLT-treated irradiated rats for 3 and 14 days (IR + MLT-3 and 14), showing a marked PCNA immuno-expression amelioration to almost normal (arrows) within the seminiferous tubules, (IHC × 100). **B** Histogram illustrating the quantification of the PCNA expression levels in various treatment groups. Values are expressed as the means ± SEM of 5 microscopic fields/ tissue samples. ** Highly significant at *P* < 0.01. ***, ###, %%%, @@@ Very Highly significant at *P* < 0.001. **& *** Significant as compared with the control 3 days group. ### Significant as compared with the control 14 days group. %%% Significant as compared with the IR 3 days group. @@@ Significant as compared with the IR 14 days group. *Cont* control, *MLT* melatonin, *IR* irradiated

The original article [1] has been corrected.
